# Clinical, Genetic, Imaging and Electrophysiological Findings in a Cohort of Patients With *GUCA1A*-Associated Retinopathy

**DOI:** 10.1167/iovs.66.2.50

**Published:** 2025-02-19

**Authors:** Gilad Allon, Siying Lin, Anthony G. Robson, Gavin Arno, Magella M. Neveu, Pirro G. Hysi, Michel Michaelides, Andrew R. Webster, Omar A. Mahroo

**Affiliations:** 1 National Institute of Health Research Biomedical Research Centre at Moorfields Eye Hospital and the UCL Institute of Ophthalmology, London, United Kingdom; 2 UCL Institute of Ophthalmology, University College London, London, United Kingdom; 3 Department of Electrophysiology, Moorfields Eye Hospital, London, United Kingdom; 4 Section of Ophthalmology, King's College London, St Thomas’ Hospital Campus, London, United Kingdom; 5 Department of Twin Research & Genetic Epidemiology, King's College London, St Thomas’ Hospital Campus, London, United Kingdom; 6 Sørlandet Sykehus Arendal, Arendal Hospital, Norway

**Keywords:** retina, guanylate cyclase activator 1A protein, human, progressive cone dystrophies, macular degeneration

## Abstract

**Purpose:**

To report findings in *GUCA1A*-associated retinopathy, a rare autosomal-dominant retinopathy.

**Methods:**

Clinical features and investigations from molecularly confirmed patients at a large referral center were analyzed (retrospective cohort study).

**Results:**

Nineteen patients (14 families), with five different variants, were included: p.(Tyr99Cys) in 10 families and p.(Leu84Phe), p.(Ile107Thr), p.(Glu111Ala), and p.(leu176Phe) in 1 family each. Mean (SD) ages at first and last visits were 38 (17) and 48 (15) years, respectively. Mean (SD) logMAR visual acuities at the first and last visits were 0.67 (0.61) and 0.94 (0.58) for right eyes and 0.63 (0.63) and 0.95 (0.74) for left eyes. Acuities ranged from 0.00 logMAR to no light perception. Most described progressive problems with central and color vision. Across 144 patient visits, logMAR acuity correlated with age (Spearman coefficients of 0.43 and 0.54 for right and left eyes, *P* < 0.001), with a high interocular correlation (coefficient 0.90, *P* < 0.001). Optical coherence tomography showed irregularity and then loss of the central ellipsoid zone. Ultra-widefield imaging showed peripheral degeneration in some patients. Electrophysiology (*n* = 13) was consistent with cone dystrophy (*n* = 11) or macular dystrophy (*n* = 2). Compared with the common p.(Tyr99Cys) variant, patients with p.(Glu111Ala) (*n* = 2) had worse vision; those with p.(Leu84Phe) (*n* = 3) were younger with earlier-onset visual loss. Patients with p.(Ile107Thr) (*n* = 2) showed later presentation, with milder acuity reduction.

**Conclusions:**

We present genotypic and phenotypic findings from the largest cohort with *GUCA1A* retinopathy. Most had progressive visual loss and electrophysiologic evidence of cone dystrophy. Possible genotype–phenotype correlations emerged, but subgroups were small for four of five variants.

The guanylate cyclase activator 1A (*GUCA1A*) gene encodes guanylate cyclase activating protein (GCAP1), a calcium-sensor protein that is highly expressed in photoreceptor outer segments. GCAP1 plays a critical role in phototransduction, where it regulates the retinal guanylyl cyclase (RetGC)–mediated restoration of cyclic guanosine monophosphate (cGMP) levels in response to a reduction in intracellular calcium concentration within photoreceptors that occurs with phototransduction.^1^

*GUCA1A* was first associated with inherited retinal disease (IRD) in 1998, when Payne et al.^2^ described a p.(Tyr99Cys) missense variant segregating with disease in a four-generation British family with autosomal-dominant cone dystrophy. Heterozygous variants in *GUCA1A* are now associated with autosomal-dominant cone, cone-rod, and macular dystrophy phenotypes,^2^^–^^7^ and association has also been reported, although considerably less frequently, with retinitis pigmentosa (RP).^8^^–^^11^ Variants in the gene are a relatively rare cause of IRD. Unlike some other autosomal IRD genes that have been associated with both dominant and recessive disease, pathogenic variants in *GUCA1A* have been reported only in autosomal-dominant disease. A US-based study of 1000 consecutive IRD cases found 7 cases with *GUCA1A* variants; the authors estimated a US-wide prevalence of 1 in 247,143 people.^8^ A Japanese study of 1192 IRD families found 3 *GUCA1A* families,^6^ a German study reported 8 of 2158 IRD patients,^12^ and a study of 800 Chinese probands with nonsyndromic IRDs (of whom 481 achieved a positive genetic diagnosis) reported 2 patients with variants in *GUCA1A*.^13^ A UK-based study of 3195 IRD families with a confirmed genetic IRD diagnosis, published from our institution in 2020, found 8 families^14^; a study from Spain, published in 2021, found 5 families with *GUCA1A*-associated disease out of a total of 3951 families who had undergone genetic testing (2100 of whom had positive molecular diagnoses).^11^ Thus, the prevalence in IRD cohorts appears to range from roughly 0.1% to 0.7%. These figures not only reflect true local prevalences but are also affected by genetic testing strategies and whether the denominator is restricted to all IRD patients/families or only those with positive molecular diagnoses.

Some studies have also provided estimates as a proportion of relevant IRD subgroups: the aforementioned Spanish study found *GUCA1A* variants accounted for 3% of families (*n* = 4) within the autosomal-dominant non-RP IRD subgroup (the fifth family was reported to have RP)^11^; a previous German study of 251 consecutive unrelated cases of macular dystrophy or cone/cone-rod dystrophy (of whom 185 had a positive genetic diagnosis found) reported 3 families with disease associated with *GUCA1A*, corresponding to a proportion of 1.2% (or 1.6% of cases with a positive molecular diagnosis).^7^

Given the relative rarity, *GUCA1A*-associated retinopathy has only been reported in small numbers of individuals to date (and several have been within studies of large IRD cohorts with minimal phenotypic details for each case), limiting our understanding of the full phenotypic spectrum and disease progression. The present study provides a genotypic and phenotypic characterization of the largest case series of patients with *GUCA1A*-associated retinopathy to date, exploring possible genotype–phenotype correlations and enabling a better understanding of the natural history of the condition.

## Methods

### Patient Ascertainment

A clinical cohort of affected individuals with IRD who have attended Moorfields Eye Hospital (London, UK) and who have been identified to harbor likely disease-causing variants in *GUCA1A* was retrospectively ascertained through a review of electronic patient records and the Moorfields inherited eye disease database.^15^ The total number of families in our cohort with confirmed IRD genetic diagnoses around the time of the search was 3954. Local research ethics approval was from Moorfields Eye Hospital and the Northwest London Research Ethics Committee, and the study was performed in accordance with the tenets of the Declaration of Helsinki.

### Clinical Phenotyping

Relevant clinical and demographic data were retrieved from electronic health records, case notes, and imaging software systems. Recorded Snellen visual acuities were converted to logMAR for statistical analysis (light perception vision documented in a single individual was assigned a value of 2.70 logMAR, and no light perception vision documented in a further individual was assigned a value of 3.00 logMAR).^16^ First, raw Spearman correlations with age were quantified (across all patient visits), and a simple linear relation was fitted to acuities as a function of age across both eyes and all patient visits. We then fitted a repeated measurements mixed model, with nested random effects for each family, patient within the family and each eye (left and right), and random intercepts for each variant using the “*lme4*” R package, following rank normalization transformation of acuities. Effects of each variant were estimated using the commonest variant as the reference.

Clinical assessment consisted of a detailed clinical history obtained from each affected individual, as well as a full medical and family history. An ophthalmic examination was performed, including assessment of visual acuity and slit-lamp biomicroscopy and fundoscopy examining the anterior and posterior segments. Imaging included spectral-domain optical coherence tomography (SD-OCT; Heidelberg Spectralis, Heidelberg Engineering, Heidelberg, Germany), ultra-widefield pseudocolour fundus photography (Optos PLC, Dunfermline, UK), and fundus autofluorescence (Optos PLC and Heidelberg Spectralis). Visual field testing was undertaken in a minority of patients (Humphrey Visual Field Analyzer; Carl Zeiss Meditec, Dublin, CA, USA).

### Visual Electrophysiology

Visual electrophysiology included pattern and full-field electroretinogram (PERG; ERG) testing, performed to incorporate the standards of the International Society for Clinical Electrophysiology of Vision^17^^–^^19^ and recorded using corneal gold foil electrodes (*n* = 12) or silver thread electrodes (*n* = 1). Additional photopic on–off ERGs were recorded from some subjects. Pattern ERG P50 peak time and amplitude were used to provide an objective measure of macular function, and full-field ERG was used to assess generalized (mainly extramacular) rod and cone system function.^19^ The ERG data were compared with a reference range derived from a control group of healthy individuals (age range, 10–79 years)^20^^,^^21^ according to the stimulus system and type of electrode used. The amplitudes of the main full-field ERG components were plotted as a percentage of the age-matched lower limit of “normal” for the dark-adapted (DA) 0.01 ERG, DA 10 ERG a- and b-waves, light-adapted (LA) 3 single-flash ERG b-wave, and LA 3 30-Hz ERG. For the latter, peak times (in milliseconds) were plotted as a difference from the age-matched upper peak time limit.

### Molecular Genetic Analysis

Genomic DNA was extracted from whole blood, and genetic testing was performed using targeted Sanger sequencing, panel-based next-generation sequencing, or whole-exome or genome sequencing. Where appropriate and available, blood samples were taken from consenting family members for familial segregation analysis. Variant classification ascribing likely pathogenicity was performed in accordance with American College of Medical Genetics and Genomics (ACMG) guidelines.^22^ Variants were annotated in relation to the most biologically relevant MANE select *GUCA1A* transcript NM_001384910.1.^23^

## Results

Nineteen individuals (a–s) from 14 families (I–XIV) with *GUCA1A*-associated retinopathy were identified, with key data listed in the Table.

**Table.** Key Demographic, Genetic, and Visual Acuity Characteristics of All 19 Affected Individuals With *GUCA1A*-Associated Retinopathy

Family	Patient	Sex	Variant (Nucleotide)	Amino Acid Change	ACMG Classification	Age of Onset, y	Age at First Visit, y	Right Eye Visual Acuity at First Visit (LogMAR)	Age at Last Visit, y	Right Eye Visual Acuity at Last Visit (LogMAR)
I	a	M	c.250C>T	p.(Leu84Phe)	LP	9	9	0.48	24	1.18
I	b	F	c.250C>T	p.(Leu84Phe)	LP	11	14	0.30	34	0.90
I	c	M	c.250C>T	p.(Leu84Phe)	LP	11	56	1.00	56	1.00
II	d	F	c.296A>G	p.(Tyr99Cys)	P	NA	28	0.60	35	0.78
II	e	M	c.296G>A	p.(Tyr99Cys)	P	5	20	0.48	25	0.30
III	f	F	c.296A>G	p.(Tyr99Cys)	P	5	32	0.78	52	1.00
IV	g	M	c.296A>G	p.(Tyr99Cys)	P	5	43	1.00	67	1.78
V	h	F	c.296A>G	p.(Tyr99Cys)	P	20	49	0.60	59	1.00
VI	i	M	c.296A>G	p.(Tyr99Cys)	P	39	39	0.18	57	1.00
VII	j	M	c.296A>G	p.(Tyr99Cys)	P	20	34	1.00	62	0.90
VIII	k	M	c.296A>G	p.(Tyr99Cys)	P	50	52	0.18	59	1.00
IX	l	M	c.296A>G	p.(Tyr99Cys)	P	30	33	0.30	37	0.60
X	m	M	c.296A>G	p.(Tyr99Cys)	P	20	27	0.48	28	0.78
XI	n	F	c.296A>G	p.(Tyr99Cys)	P	20	51	0.18	54	0.11
XII	o	F	c.320T>C	p.(Ile107Thr)	P	25	68	0.18	70	0.48
XII	p	M	c.320T>C	p.(Ile107Thr)	P	74	74	0.30	74	0.30
XIII	q	M	c.332A>C	p.(Glu111Ala)	LP	23	44	Light perception	52	Light perception
XIII	r	M	c.332A>C	p.(Glu111Ala)	LP	20	26	1.48	40	1.40
XIV	s	M	c.526C>T	p.(Leu176Phe)	LP	28	29	0.60	37	0.60

F, female; LP, likely pathogenic; M, male; NA, information not available; P, pathogenic.

### Demographics and Clinical Characteristics

The mean (SD) age at the first visit was 38 (17) years (range, 9–74 years), with two individuals (I-a and I-b) reviewed in childhood before age 16 years. The mean age at the final visit was 48 ± 15 years, with a mean (SD) follow-up duration of 10.3 (8.6) years. Thirteen individuals were male (68.4%) and six were female (31.6%). Eight individuals were of White British ethnicity (42.1%,) and one individual was of Asian Pakistani ethnicity (5.3%); for the remaining 10 individuals, their ethnicities were unrecorded. Most individuals had no other ocular pathology documented. For individual VIII-k, bilateral glaucomatous optic neuropathy was suspected. One patient (IV-g) developed a Coats-like reaction in the left eye that developed into an exudative retinal detachment despite laser treatment, resulting in a blind left eye.^24^

**Figure 1.**
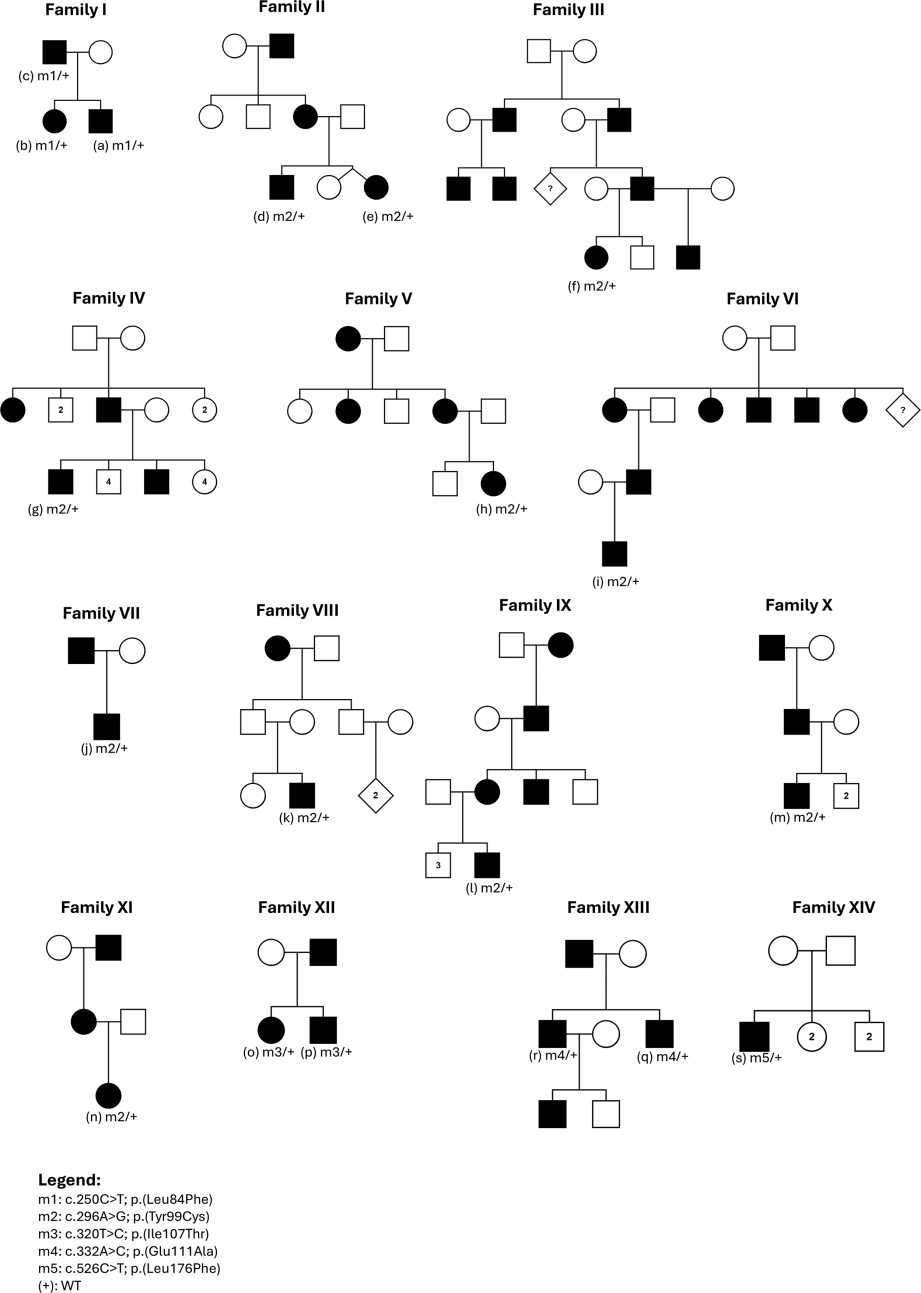
Pedigrees for the 14 families with variants shown for each individual who underwent genetic testing.

For 13 of 14 families, a history of affected individuals in multiple generations was elicited. In one family (XIV), there was no history of affected individuals in other generations. Pedigrees are shown in Figure 1.

The mean (SD) age of onset of symptoms was 23 (17) years (range, 5–74 years). Thirteen patients (68.4%) described poor color discrimination as their initial symptom and four patients (21.1%) reported initial central vision loss, one patient (5.3%) described photophobia, and one patient (5.3%) was unable to recall their initial symptoms. Overall, across the entire duration of follow-up, 17 patients (89.5%) reported central vision deficits, 16 patients (84.2%) described color vision deficits, 15 (78.9%) reported some degree of photophobia, 1 patient (IX-l) noted nyctalopia (5.3%), and another patient (VIII-k) described metamorphopsia (5.3%).

**Figure 2.**
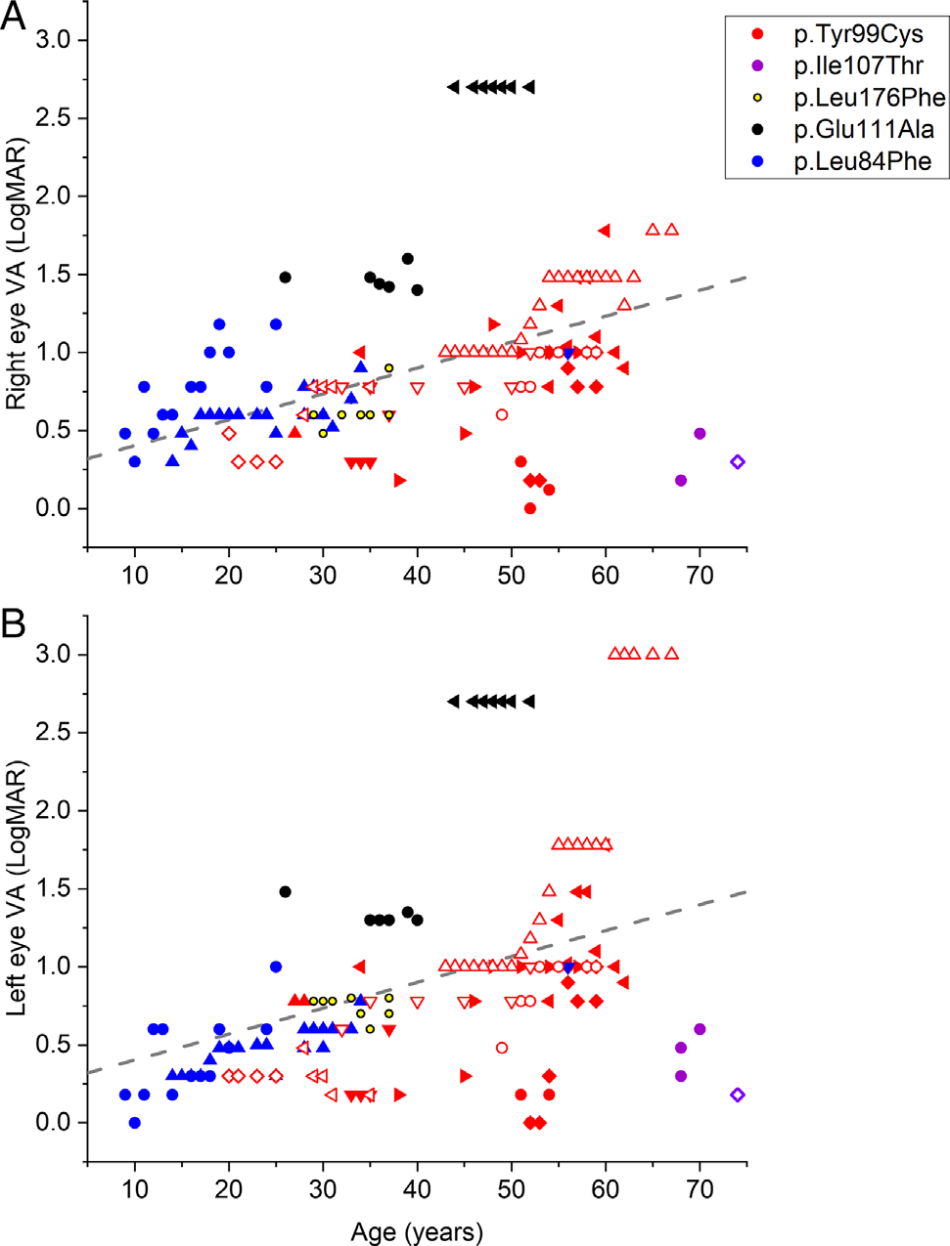
Visual acuities across 144 patient visits plotted against age for right eyes (**A**) and left eyes (**B**). Each color denotes a different genetic variant (shown in legend in *upper panel*). Within each color group, each symbol denotes an individual patient. The *dashed line* shows a simple linear fit (fitted across all patient visits and both eyes). LogMAR acuity correlated positively with age, indicating worse visual acuities in older patients overall, although several exceptions are seen. Findings from a mixed-effects model are described in the text.

Visual acuities are plotted against age at baseline and across the follow-up period (144 patient visits) in Figure 2. Different colors represent different genetic variants, and within each color group, different symbols represent different individuals. The dashed lines plot a simple linear fit across all visits (and both eyes). A significant correlation was seen between visual acuity and age, with worse acuity on average at older ages; Spearman correlation coefficients (between logMAR visual acuity and age) were 0.43 and 0.54 for right and left eyes, respectively (*P* < 0.001). Interocular correlation was high (coefficient 0.90; *P* < 0.001).

Mean (SD) visual acuities at first and last visit were 0.67 (0.61) and 0.94 (0.58) for right eyes and 0.63 (0.63) and 0.95 (0.74) for left eyes. For the commonest variant, p.(Tyr99Cys), affecting 11 patients, mean (SD) ages at first and last visits were 37 (11) and 48 (15) years: mean (SD) visual acuities for these patients at first and last visit were 0.52 (0.31) and 0.82 (0.43) for right eyes and 0.47 (0.34) and 0.88 (0.77) for left eyes.

Fitting a mixed model (see Methods) across all visits, the effect of age remained significant (*P* = 0.00023). The effect size could not be quantified precisely as data had been transformed by rank normalization, but it corresponded roughly to a worsening of approximately 0.20 logMAR per decade. In addition, the model suggested that the p.(Ile107Thr) variant showed less severe reduction in acuity compared with the p.(Tyr99Cys) variant (*P* = 0.0022) and that male sex was associated with worse acuity (*P* = 0.023).

Refractive data were available for 14 patients; the mean (SD) spherical equivalent was –0.80 (4.1) diopters (D) (range, –12.5 to +4.5 D) for the right eye and –0.25 (3.6) D (range, –8.0 to +6.5 D) for the left eye.

**Figure 3.**
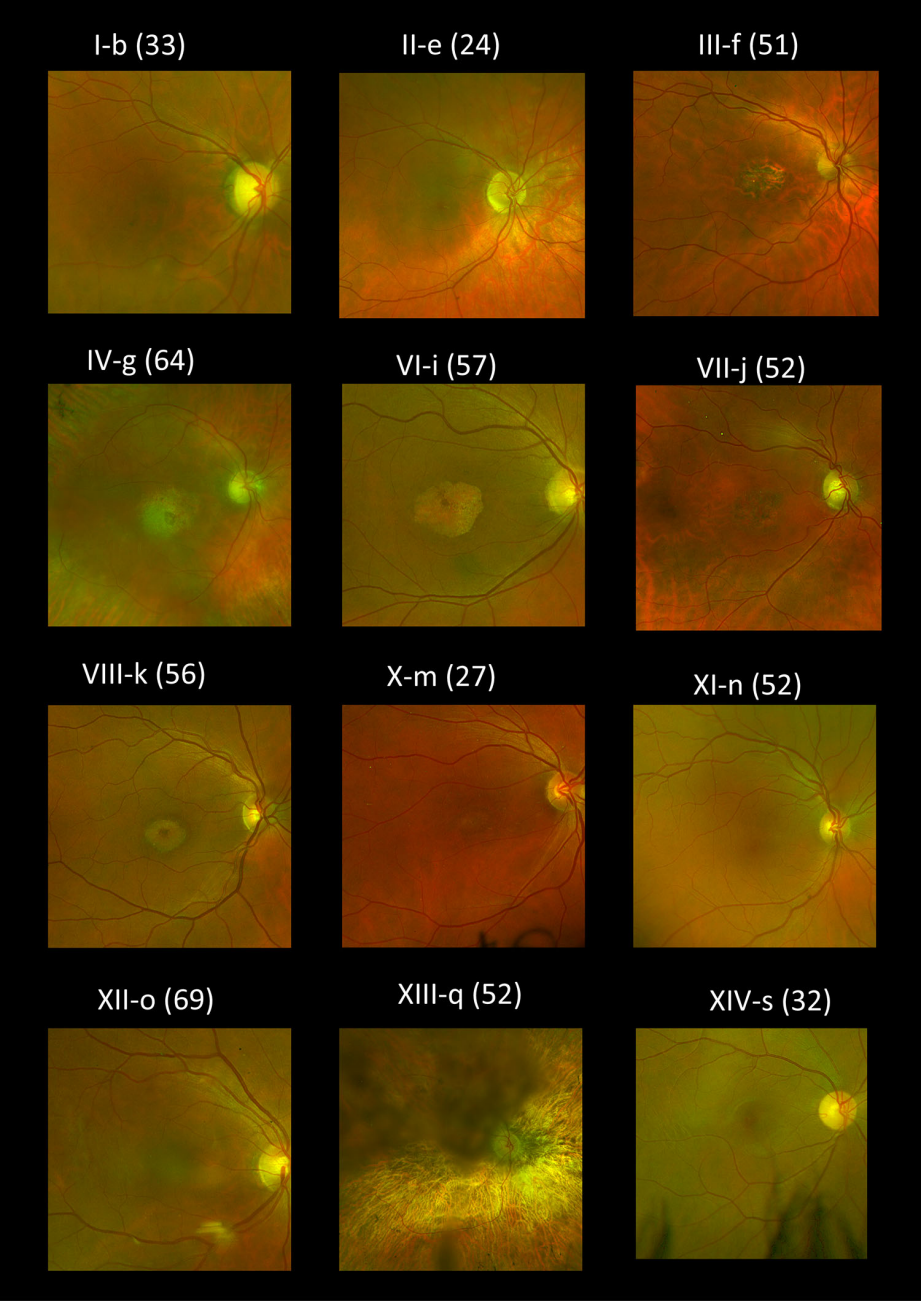
Right eye pseudocolor fundus imaging for patients where available. The age at examination is denoted in parentheses.

**Figure 4.**
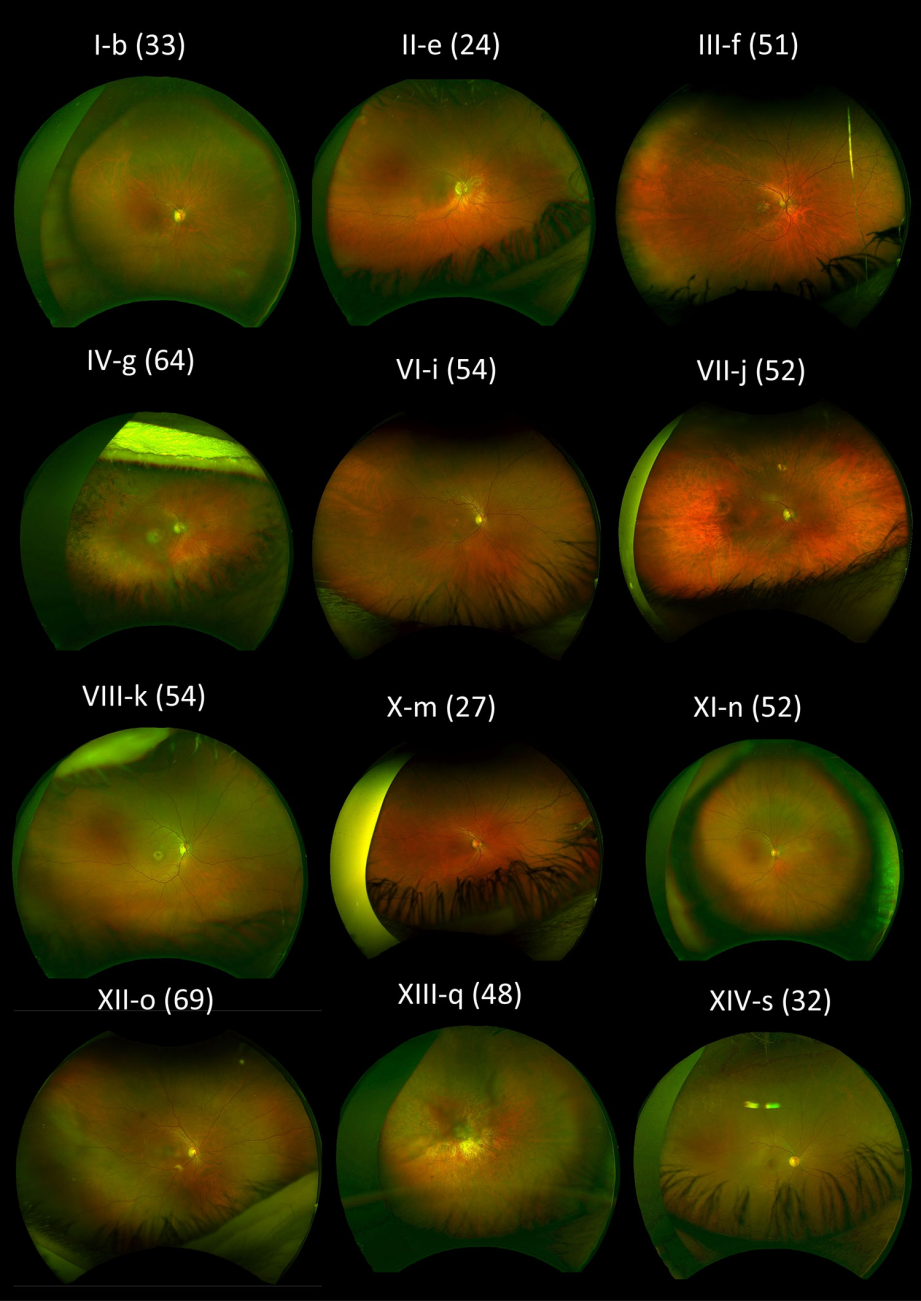
Right eye ultra-widefield pseudocolor fundus imaging for patients where available. The age at examination is denoted in parentheses.

**Figure 5.**
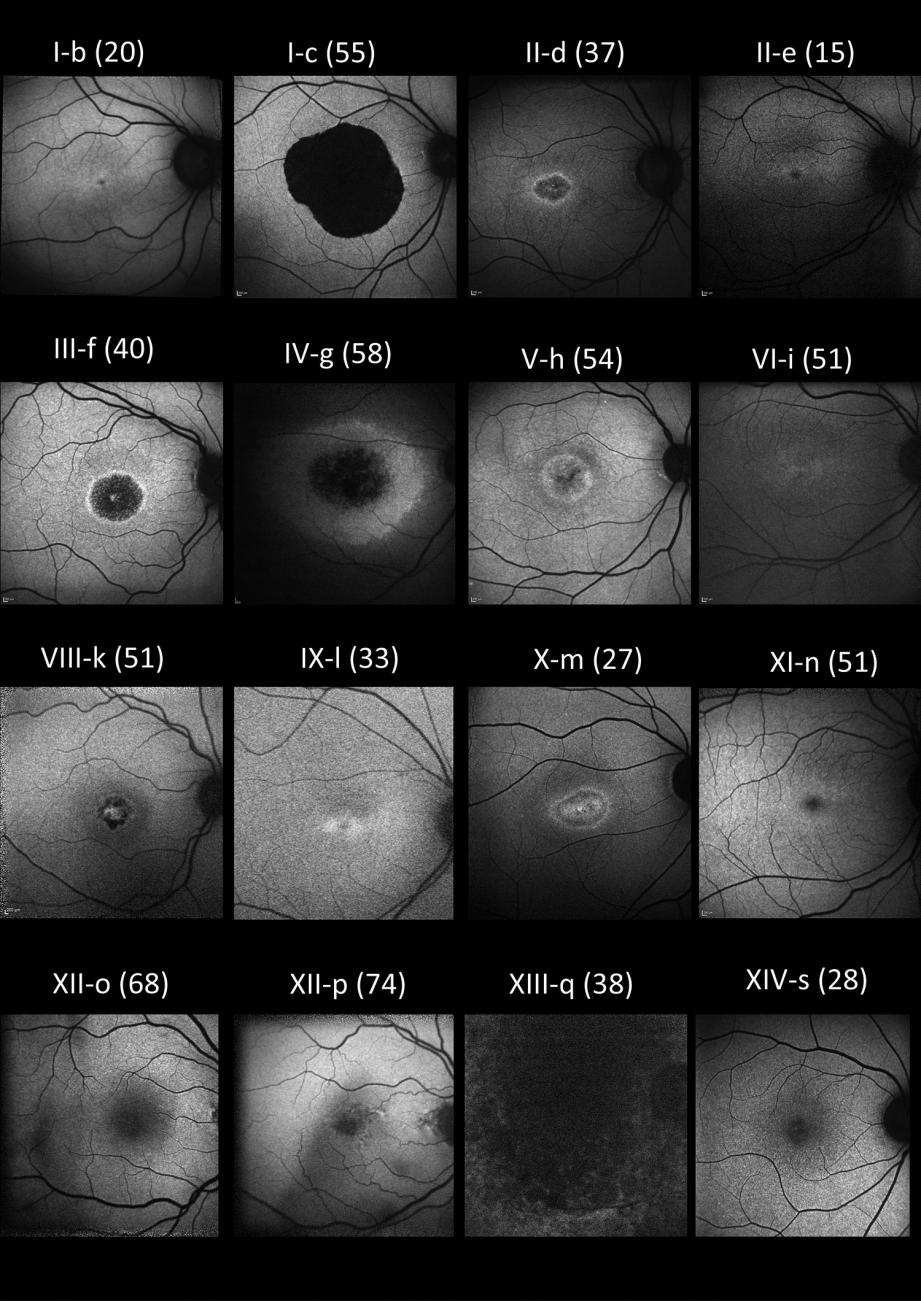
Right eye fundus short-wavelength (488 nm) autofluorescence imaging for all patients where available. The age at examination is denoted in parentheses.

**Figure 6.**
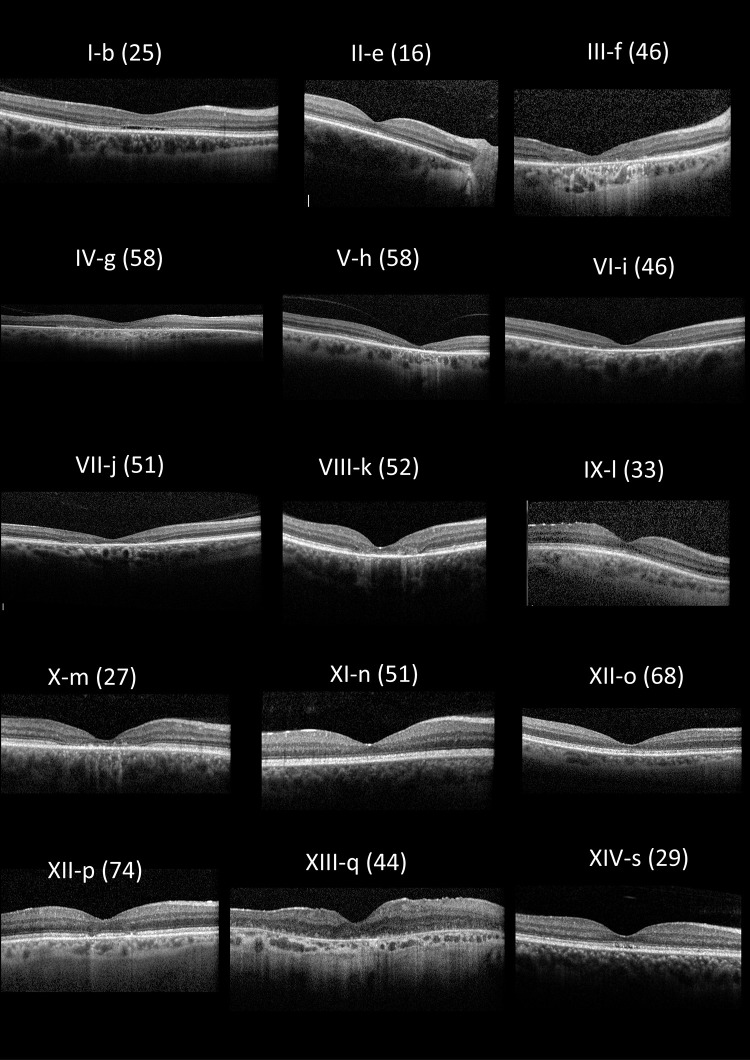
Right eye optical coherence tomography imaging for all patients where available. The age at examination is denoted in parentheses.

Fundus appearances and retinal imaging revealed a high degree of interocular symmetry in general. Representative retinal imaging is shown in Figures 3 to 6: Figures 3 and 4 depict ultra-widefield pseudocolor images (Fig. 3 focuses on the macula and Fig. 4 shows peripheral findings); Figure 5 shows short-wavelength (488 nm), macular autofluorescence images; and Figure 6 shows macular SD-OCT images. The scans shown are from the first visit with available clear images from the relevant investigation.

**Figure 7.**
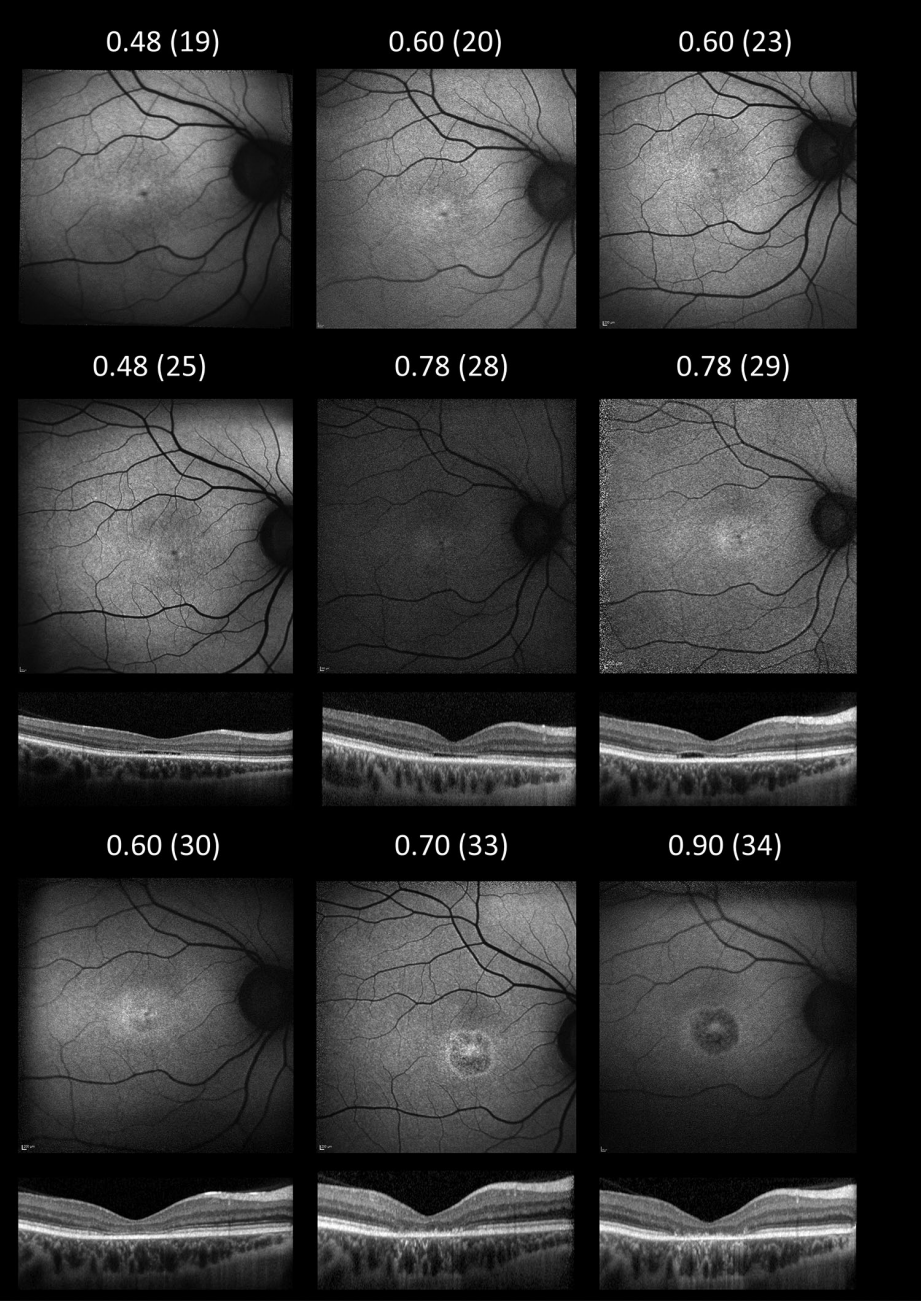
Longitudinal multimodal imaging showing visual acuity measurements, fundus autofluorescence, and optical coherence tomography findings in a single individual (I-b) across a 15-year follow-up period. The age at examination is denoted in parentheses. OCTs were available from age 25.

Maculopathy was evident in all individuals on OCT imaging. Partial ellipsoid zone (EZ) disruption was seen (mean [SD] age 42.5 [18.0] years; range, 17–74 years) or complete loss of the EZ layer (mean [SD] age 44.2 [13.4] years; range, 26–58 years). No individuals were noted to have cystoid macular edema or choroidal neovascularization. Visual field tests, where performed (four patients), demonstrated central and paracentral scotomas. Multimodal longitudinal imaging in a single individual (I-b) highlighting progressive changes in visual acuity, fundus autofluorescence, and OCT over a period of 15 years is shown in Figure 7.

### Visual Electrophysiology

**Figure 8.**
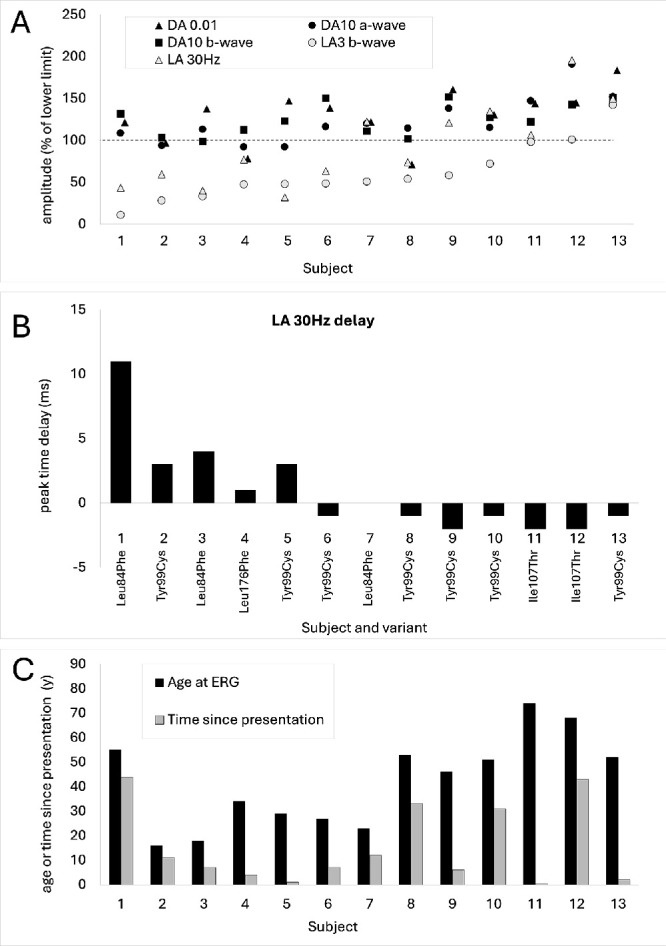
Full-field ERG parameters in 13 subjects tested according the International Society for Clinical Electrophysiology of Vision standard methods. (**A**) The amplitudes of the DA 0.01 ERG, DA 10 ERG a- and b-waves, LA 30-Hz ERG, and LA 3 ERG b-wave are plotted as a percentage of the age-matched lower limit of the (“normal”) reference range (*horizontal broken line*), with values arranged in ascending order of the cone-mediated LA 3 ERG b-wave amplitude for clarity. Data points for DA 0.01 ERGs are plotted slightly to the right of other data for clarity. (**B**) The LA 30-Hz peak times as a difference from the age-matched upper limit of normal timing with corresponding *GUCA1A* variants highlighted. (**C**) The age of the patients at the time of testing and time since presentation. Patient numbering is consistent in all three panels.

**Figure 9.**
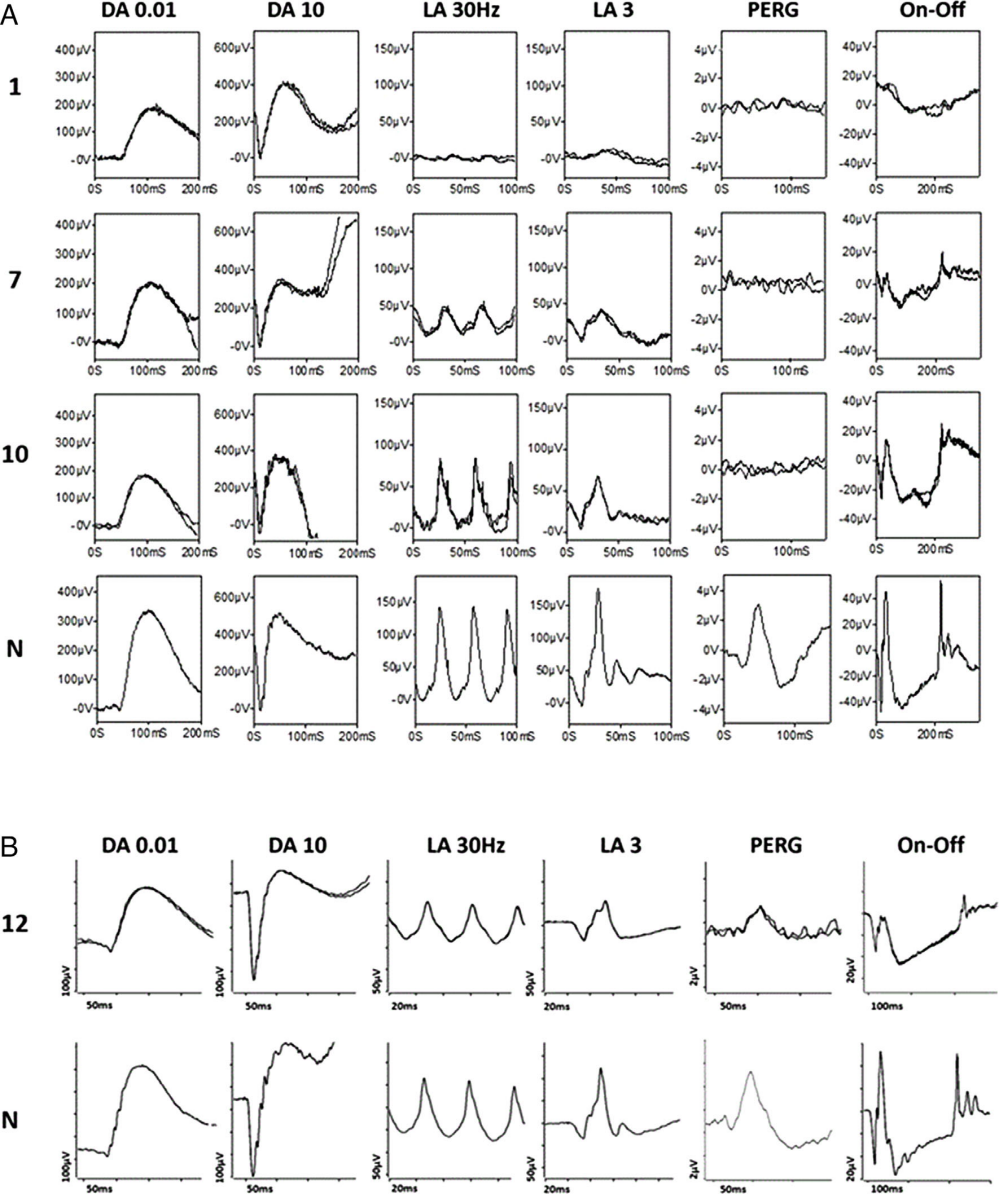
Representative International Society for Clinical Electrophysiology of Vision standard full-field and pattern ERGs. (**A**) Recordings from ERG patients 1 (aged 55 years), 7 (23 years), and 10 (51 years) corresponding to the patient numbering used in Figure 8. Representative control (“normal”) recordings are shown for comparison (N). Data are shown for the right eyes only, as all showed a high degree of interocular symmetry. Patient traces are superimposed to demonstrate reproducibility. (**B**) Recordings from patient 12, who was tested using different equipment, and single-flash and PERG recordings include a 20-ms prestimulus delay. There is ERG evidence of a severe (patient 1), moderate (patient 7), and mild (patients 10 and 12) cone dystrophy. Undetectable PERG P50 components are consistent with severe macular involvement, except in patient 12, who showed a normal PERG P50 in keeping with spared macular function.

**Figure 10.**
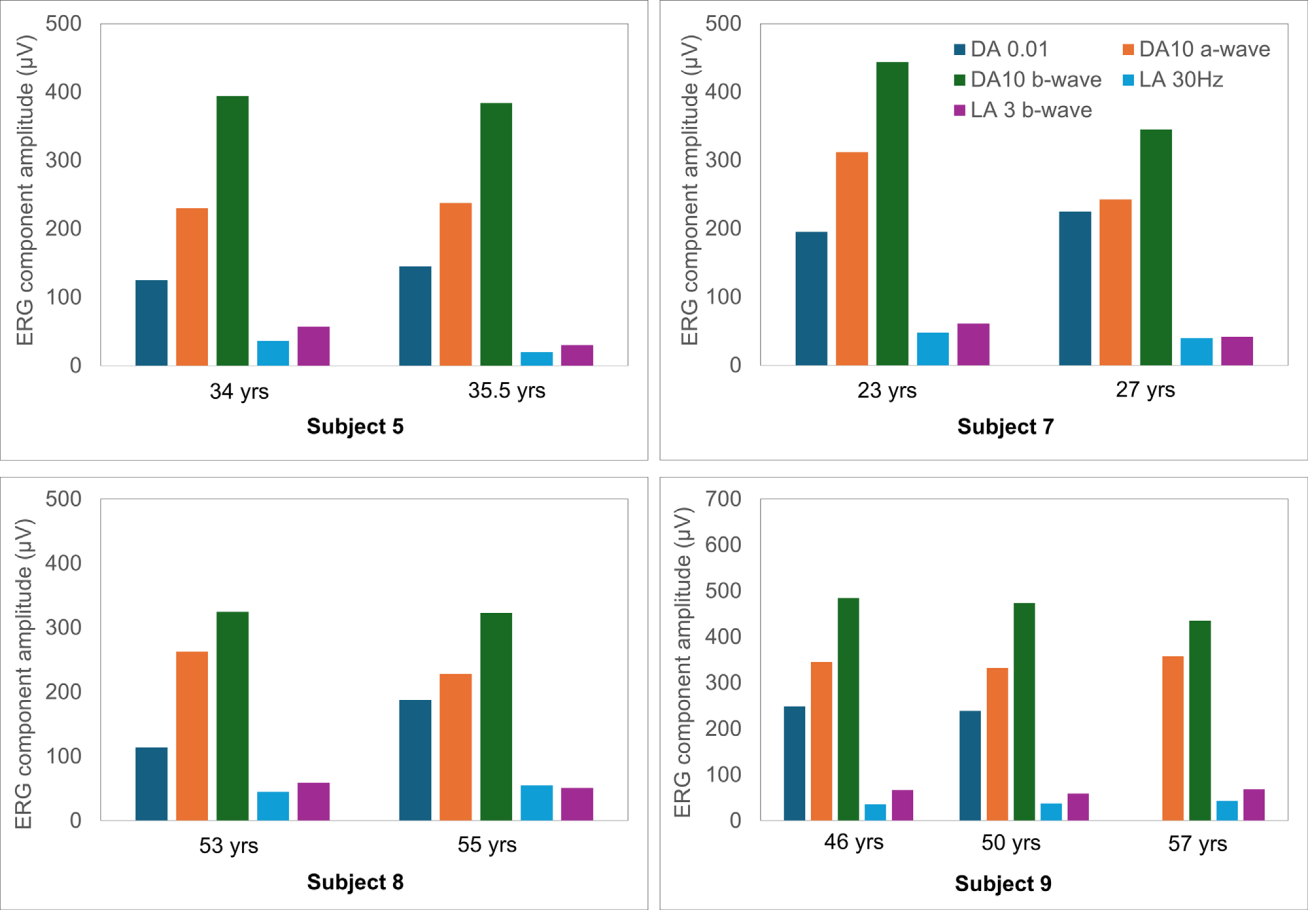
Comparison of the main ERG component amplitudes at initial testing with those obtained at follow-up for four patients. These are ERG patients 5 (*top left*), 7 (*top right*), 8 (*bottom left*), and 9 (*bottom right*), monitored over 1.5, 4, 2, and 11 years, respectively. Ages at the time of testing are shown on the x-axis. Final follow-up DA 0.01 ERGs in case 9 were distorted by eye movement and are excluded. LA 30-Hz ERG peak times were stable in all four individuals (data not shown).

ERGs were available from 13 patients, and relevant data are shown in Figures 8 to 10. (Patients who underwent ERGs are numbered 1 to 13 in these figures.) Figure 8 compares the main DA and LA full-field ERG component amplitudes and LA 30-Hz flicker peak times for each of the 13 subjects tested and shows the age at the time of testing and time since presentation. Figure 9 shows representative recordings. Figure 10 shows ERG amplitudes for four patients who underwent testing on more than one occasion.

ERG findings were consistent with cone dystrophy in 11 subjects and with macular dystrophy in 2 cases (with normal full-field ERGs; these were subjects 11 and 13 in Fig. 8A). Ten of 13 subjects had subnormal LA 30-Hz or LA 3 (single-flash) ERGs (Fig. 8A), and there was an additional LA 30-Hz ERG delay in the 5 with the smallest LA responses (Fig. 8B; delay 1–11 ms). In one other individual, the LA 3 ERG b-wave was borderline, but the LA 3 and on–off ERGs had subnormal b/a ratios (Fig. 9B; ERG case 12; sister of case 11). In most, the LA 3 ERG b/a ratio was less than (*n* = 8) or similar to (*n* = 2) the fifth percentile of the control group. Photopic on–off ERGs showed a subnormal (*n* = 2), a borderline (*n* = 2), or an electronegative waveform (*n* = 2 including case IX-l) in the eight subjects who underwent testing.

The 10 subjects with clearly subnormal LA ERGs included 9 with symptoms of photophobia, also reported in case 11. Two patients without photophobia had borderline or normal-amplitude LA full-field ERGs, including one of two subjects with a detectable (and normal) pattern ERG (Fig. 9B; case 12), consistent with spared macular function. The PERG P50 was of borderline amplitude in the only other case with a detectable response (case 11).

Rod system–specific dim flash (DA 0.01) ERGs were truncated by blink artifacts in two subjects and were normal in all but one subject, the latter attributed to incomplete mydriasis and reduced retinal illumination (pupil diameter 6 mm; Fig. 8, case 8). Minimal reductions in the mixed rod- and cone-mediated DA 10 ERG a-waves were seen in three subjects with moderate to severe cone system dysfunction, but these reductions were likely due to loss of the dark-adapted cone system contribution (rod system–selective DA 0.01 ERGs were normal).

### Molecular Genetics

Heterozygous missense variants in *GUCA1A* were identified in all 19 individuals in our study cohort (Table). All variants identified were classified according to ACMG guidelines as pathogenic or likely pathogenic, and all variants have been previously published,^2^^,^^7^^,^^9^^,^^12^^,^^13^^,^^25^^–^^27^ although not all previous reports included full phenotypic information.

## Discussion

This study describes the largest cohort of patients with *GUCA1A*-related retinal dystrophy to date. Clinical, structural, and functional phenotypes, including longitudinal assessments of visual acuity, retinal imaging, and electroretinography, are detailed in 19 individuals from 14 unrelated families (representing approximately 0.35% of our cohort of families with genetically confirmed IRD diagnoses). The patients harbored five different pathogenic missense alleles in *GUCA1A*, allowing for genotype–phenotype relationships to be tentatively explored. Comprehensive visual electrophysiologic measures of macular and retinal function are quantified in patients of widely differing ages, expanding on the phenotypic spectrum.

The p.(Tyr99Cys) variant was the most commonly encountered *GUCA1A* variant in our study and was identified in 11 individuals from 10 families, accounting for over half of all affected individuals with *GUCA1A*-associated retinopathy within our study cohort (and over 70% of the families). This variant was first described in the *GUCA1A* discovery article by Payne et al.,^2^ where it was identified segregating with autosomal-dominant cone dystrophy in a four-generation British family. It has subsequently been identified in British, US, and Chinese IRD cohorts,^9^^,^^13^^,^^27^ and two other substitutions affecting the same codon have been reported in a Japanese cohort.^6^

Within our study cohort, commonly reported subjective complaints included central vision loss, poor color vision discrimination, and photophobia. The most frequent presenting complaint was color vision impairment. An overall decline in visual acuity was noted over the duration of follow-up, with a significant correlation seen between age and visual acuity, denoting worsening acuity with age. The average age of onset of symptoms was 23 years, although there was a wide range from 5 to 74 years. Visual deterioration appears to be gradual, and vision may rarely remain relatively preserved beyond the seventh decade (Fig. 2).

Most families reported vision impairment affecting individuals in more than one generation (Fig. 1), consistent with autosomal-dominant inheritance. For one family (VIII), the proband's parents were reportedly unaffected, but the paternal grandmother was reported to have been registered blind. For family XIV, there appeared to be no one else affected other than the proband. In both cases, the parents were unavailable for examination or genetic testing to establish whether these were cases of incomplete penetrance or whether the variant had arisen de novo.

Despite the limited size of certain variant subgroups, there seem to be suggestions of genotype–phenotype correlation. Compared with the common p.(Tyr99Cys) variant, patients with p.(Glu111Ala) (*n* = 2) had worse vision. There has been no prior report of the specific phenotype associated with this variant. Within our cohort, patients with p.(Leu84Phe) (*n* = 3) were younger with an earlier onset of visual loss. This is consistent with findings from an earlier study reporting a large three-generation Spanish family with autosomal-dominant retinal dystrophy due to *GUCA1A* p.(Leu84Phe), where all affected individuals experienced early-onset visual loss within the first two decades of life with rapid progression.^26^ Patients with p.(Ile107Thr) within our study cohort (*n* = 2) demonstrated a relatively delayed presentation, with mild reduction in acuity relative to age; here, the difference (compared with patients with the p.(Tyr99Cys) variant) emerged as statistically significant in our mixed model. To date, there has only been a single previous report detailing the clinical characteristics of a single individual with retinal dystrophy associated with this variant. This individual experienced loss of central vision at age 36 years, with visual acuities maintained at 0.6 to 0.7 decimal (equivalent to approximately 0.2 logMAR) at 38 years of age.^26^ All of the inter-genotype comparisons should be taken with caution given the small numbers. Even for the p.(Tyr99Cys) variant, a range of severities was observed, indicating the existence of likely modifiers yet to be identified.

Disease-associated variants cluster in specific domains of the protein, particularly EF-hands 3 and 4, affecting calcium sensitivity, with possible effects on dimerization and interaction with other proteins.^28^^–^^35^ The encoded wild-type protein, GCAP1, is inhibited by calcium binding (intracellular calcium levels are higher in the dark), and this inhibition is relieved as calcium levels fall during the photoreceptor light response. Several *GUCA1A* disease-associated variants render the protein less sensitive to calcium inhibition; thus, it continues to activate guanylyl cyclase even in the dark, leading to supranormal levels of cGMP. As only specific protein changes will have this effect, this might explain the relative rarity of *GUCA1A*-associated disease compared with other IRDs. A particular variant, p.(Pro50Leu), was reported in 2001 to be associated with disease^4^; this variant was quite distant from other disease-associated variants, and studies showed no effect on calcium sensitivity. Later, disease in the reported family was in fact shown to be attributable to a pathogenic variant in *RPGR*, and so the p.(Pro50Leu) variant is likely a benign polymorphism.^36^ It is likely that even among pathogenic variants, disease severity may vary, and future larger studies will be better powered to explore such genotype–phenotype correlations.

Sex has been shown to be a possible modifier in some autosomal macular dystrophies,^37^^–^^39^ and it is interesting that we found male sex to be associated with worse visual acuity. However, given the small numbers, larger studies would again be needed to explore whether this is a true association.

In general, there was a correlation observed between a patient's visual acuity and symptoms, as well as with imaging findings documented on fundus photography, autofluorescence, and OCT. This is demonstrated in Figure 7, depicting progressive involvement on imaging and visual acuity decline as the disease progresses in individual II-b. This figure demonstrates the presence of an “optical gap” between the neurosensory retina and the retinal pigment epithelium (RPE), with loss of the ellipsoid zone, at the early stages of the disease in this patient, which eventually evolves into complete central outer retinal atrophy of the macula over time.

Fundus autofluorescence demonstrated high sensitivity in detecting maculopathy and in some cases was able to identify macular involvement before findings were apparent on clinical examination or color or pseudocolor fundus images, although the OCT was usually abnormal. Figure 5 shows the spectrum of autofluorescence appearances. The findings are not specific to this disorder and can resemble the imaging appearances seen in several other macular or cone dystrophies as well as cone dysfunction syndromes.^40^ Peripheral involvement with pigment spicules was noted in two individuals (Fig. 4): IV-g, who was heterozygous for p.(Tyr99Cys), and XIII-q, who was heterozygous for p.(Glu111Ala). In the latter patient, the nature and progression of symptoms were still more in keeping with a cone-rod, rather than a rod-cone, dystrophy, and other family members did not show the pigmentary changes. *GUCA1A* variants have uncommonly been described in association with retinitis pigmentosa^2^^,^^8^^–^^11^ (with little phenotypic detail in some of those studies as they were summary reports from large cohorts). This is the first reported instance of the p.(Glu111Ala) variant being associated with peripheral pigmentary change. Most cases in the present cohort and in the literature are consistent with a macular or cone dystrophy phenotype, and so in patients in whom the clinical history is more in keeping with a rod-cone dystrophy, the possibility of alternative genetic causes should be considered.

Figures 6 and 7 show that OCT imaging can also be a useful indicator of disease severity, with central atrophy correlating with visual acuity and symptoms. The changes observed on OCT scans begin with mild EZ irregularity, progressing to atrophy affecting outer retinal layers, including the outer nuclear layer, and RPE irregularity. In addition to the mild EZ irregularity typically observed in the early stages of the disease, partial EZ disruption was also observed as the disease progressed and visual acuity levels reached approximately 0.3 logMAR. More complete loss of the photoreceptor layer was subsequently noted years later when visual acuity fell below 0.7 logMAR.

The “optical gap” OCT feature has been depicted previously in *GUCA1A*-associated disease^41^ and in several other IRDs. Oh et al.^42^ published findings in 36 patients showing this feature in 2020: associated genes in that cohort were *ABCA4*, *CNGA3*, *CNGB3*, *ATF6*, *PDE6C*, *RP1L1*, *GUCA1A*, *GUCY2D*, *PRPH2*, *RAB28*, and *PITPNM3*. The sign has also been reported in blue cone monochromacy^43^ and in disease related to other genes, including *KCNV2*,^44^ *SCA7*,^45^ *POC1B*,^46^ and *MFSD8*.^47^ Nonhereditary causes also exist, including retinopathy associated with tamoxifen, Poppers, vitreomacular traction, macular telangiectasias, central serous chorioretinopathy, and photic damage (including laser and solar retinopathy), as summarized by Oh et al.^42^ in their discussion. A common feature of many of these disorders is primary involvement of cones (or specifically foveal cones), particularly the outer parts of the photoreceptor, and so it is possible that selective degeneration at this level, in combination with preservation of other cellular structures that maintain outer retinal structural integrity, gives rise to this OCT appearance; in some cases, subsequent disease progression might then lead to “collapse” of outer retinal structure, obliterating the optical gap.

In conclusion, this study presents the largest series of patients with *GUCA1A*-associated retinopathy to date, providing insights into the spectrum of clinical manifestations of the disease. Common symptoms include central vision loss and deficits in color vision, with a noticeable trend toward worsening visual acuity with age. There is considerable variation in the age of symptom onset and disease severity. The p.(Leu84Phe) variant appears to be associated with an earlier onset of vision loss; the p.(Ile107Thr) might be associated with later presentation and milder loss of acuity. Structural anomalies are first detected at and predominantly affect the outer photoreceptor layer, consistent with patterns of gene expression. While this condition primarily affects the central macula, cases with peripheral involvement and pigment spicules have been described. Electrophysiology shows signs of cone dystrophy without any clear rod involvement in those patients tested; this might be related to the test being performed before the onset of marked peripheral involvement. Interestingly, some of the mildest ERG phenotypes were observed among the oldest patients with heterogeneous genotypes, suggesting the influence of additional modifiers on disease progression.
